# Therapists’ Expressions of Agreement in Therapeutic Conversations With Chinese Children With ASD: Strategies, Sequential Positions and Functions

**DOI:** 10.3389/fpsyg.2021.792167

**Published:** 2022-01-10

**Authors:** Xiaorong Zeng, Bosen Ma, Chenxi Li, Laiyun Zhang, Chenxi Li, Haifeng Li

**Affiliations:** ^1^School of Foreign Languages, Jiangxi Agricultural University, Nanchang, China; ^2^School of International Studies, Zhejiang University, Hangzhou, China; ^3^Department of Rehabilitation, The Children’s Hospital, Zhejiang University School of Medicine, Hangzhou, China

**Keywords:** agreement expression, therapist, sequential position, strategy, function, Chinese children with ASD

## Abstract

Based on conversations between 10 Chinese children with Autism Spectrum Disorders (ASD) and five therapists in the context of Naturalistic Intervention, this study investigated the therapists’ agreement expressions in this typical setting. The study found that (1) the therapists mainly used four agreement strategies: acknowledgment, positive evaluation, repetition and blending. These four strategies could be used individually or in combination. The first three strategies and their combinations were used frequently during the therapeutic conversation. (2) With the major occurrences in the post-expansion position, the agreement expressions in the therapeutic conversation mainly performed three functions, namely, creating a supportive therapeutic relationship, serving as positive reinforcers and implementing interventions pertinent to communication skills. (3) This study proposed that the therapists’ preferred use of agreement expressions in the intervention process could be explained by the features of Naturalistic Intervention.

## Introduction

Autism Spectrum Disorder (ASD) is a developmental disorder which is defined as “having difficulties in social communication, as well as restricted and repetitive behavior, interests or activities” (American Psychiatric Association, 2013, p. 50). While the existing estimates are variable, the prevalence rate of ASD is believed to be increasing over time (Elsabbagh et al., 2012). In the United States, the prevalence of ASD among children aged 8 years across 11 surveillance sites was 18.5 per 1,000 (1 in 54) in 2016, an increase of 9.5% compared with figures recorded in 2014 (Maenner et al., 2020). In mainland China, the prevalence of autism among children between the ages of 6 and 12 years in 2018 was estimated to be 7 per 1,000 (1 in 142) and the population of this group is as large as 700,000 to one million (Zhou et al., 2020).

To improve the life quality of this increasing population with a disability, early diagnosis and intervention are believed to be necessary and effective. During the process of intervention, the interpersonal interaction between the therapists or parents and the individuals with ASD is significant, as the intervention is implemented mainly through interaction. This significance is supported by previous studies on psychotherapy interaction, doctor-patient interaction and nurse-patient interaction (Lambert and Bergin, 1994; Karpiak and Benjamin, 2004; Wu, 2019, 2021; Demiris et al., 2020; Peoples et al., 2020; Taylor and Doolittle, 2020). These studies have revealed that common factors such as warmth, attention, empathy, affirmation and understanding play a positive role in client improvement. Due to the proven significance of therapeutic interaction, more qualitative and quantitative research efforts are needed to explore the forms, functions and the effectiveness of various interaction strategies employed by therapists when interacting with clients suffering from different disorders. For this reason, the study probed into one common yet under-investigated linguistic phenomenon in therapeutic conversations with Chinese children with ASD, that of the therapist’s use of agreement expressions.

As a common phenomenon in daily interaction, agreement has been defined as approval of a speaker’s opinions (Stenstrom, 1994) or as the willingness to accept the proposals and propositions of others (Eggins and Diana, 2005) or as a show of support by one speaker for the beliefs or propositions expressed by another (Johnson, 2006). Previous studies on verbal agreement in interaction have explored various aspects of agreement, including the strategies of expressing agreement, the sequential organization in which the expressions occur, the functions of agreement, and the factors influencing the selection of agreement or disagreement. Considering the research focus of the current study, we reviewed the research concerning the first three aspects.

In existing studies, the strategies of expressing agreement were classified as either form or content. As for forms, Heritage and Raymond (2005) examined four types of strategies through which a second speaker can index agreeing assessments with that of a first speaker: repeat/confirmation + agreement token; “oh”-prefacing; tag questions and negative interrogatives. In terms of content, Pomerantz (1984) classified agreement into “upgraded” (strong agreement), “same level” and “downgraded” (weak agreement). The “upgraded” expression is either a stronger evaluative term than the prior term or a term in which an intensifier is added to modify the prior evaluative term. As for the “same level” agreement expression, the previously used evaluative term may be repeated and may often include the word “too.” The “downgraded” expression refers to a weaker evaluative term than the previous term and could be followed by a disagreement expression. Bercelli et al. (2008) focused on clients’ strategies of response to therapists’ formulation and reinterpretation of their expressions and discussed two strategies of agreement: mere agreement and extended agreement. The former refers to the agreement tokens with no other elaborations, while the latter refers to the expressions in which the client provides an explanation by offering evidence in a narrative or non-narrative form.

Regarding the features of sequential organization of agreement, the most acclaimed research can be found in Pomerantz (1984). Focusing on the overall features of sequential organization of agreement in terms of expressing assessments, Pomerantz found that the agreement components were usually performed with a minimal gap between the turn taken by the first person in the conversation and the turn of the subsequent individual providing the agreement; the agreement was the preferred next action across most initial assessments. However, if the initial assessment was produced with self-deprecating sentences, the formulation of an agreement with prior self-deprecation was not usually preferred. In this case, the agreements could be achieved with weakly stated agreement components.

Various strategies of agreement perform different functions in conversation. For instance, through mere agreement and extended agreement (Bercelli et al., 2008), clients could show their understanding and agreement with therapists’ reinterpretations. Moreover, the client could display a change of perspective in relation to his/her own events or experiences and present this as if triggered by the therapists’ utterances. As for the four types of agreement discussed in Heritage and Raymond (2005), repeat/confirmation + agreement token and “oh”-prefacing conveyed the second speakers’ agreement as well as the epistemic independence or their assessment, while tag questions and negative interrogative strategies upgraded the agreement by conveying that the second speaker had a claimed epistemic right over the first speaker with regard to the issue. In Chinese conversation, the agreement expressions like “*hao*” and “*dui*,” served two functions: linking functions and positive response functions. In terms of linking functions, “*hao*” is often used as a marker of closure or transition indicating discourse boundaries, while “*dui*” marks the continuity of conversation. As for the positive response functions, “*hao*” is used to express acceptance of the other speaker’s act or utterance, whereas “*dui*” conveys acknowledgment of the propositional content of the utterance produced by other speakers (Wang et al., 2010).

Most of the studies on agreement were conducted using the conversation data of naturally occurring daily conversation (Pomerantz, 1984; LoCastro, 1986) or computer-mediated discussion (Baym, 1996); few studies analyzed the strategies and functions of agreement expressions produced by clients in therapeutic conversation (Bercelli et al., 2008). Much remains unknown as to the way in which therapists use agreement expressions as well as the functions agreement expressions perform in therapeutic conversation with ASD individuals. Moreover, most previous research on psychotherapeutic conversation with autistic individuals has explored either discursive issues, such as the construction of autism identity in conversation (Nadesan, 2005, 2008; Lester, 2014, etc.) or the conversational competence and impairment of autistic individuals (Damico and Nelson, 2005; Stiegler, 2007; Korkiakangas and Rae, 2014; Wiklund, 2016; Stickle et al., 2017; Wiklund and Laakso, 2020, etc.), while fewer studies have investigated the therapists’ conversational strategies, although it is believed that conversation or talk is “at the nucleus of psychotherapeutic practice” (Pamela, 2013, p. 4).

Proceeding from the existing research, the current study aimed to enhance the investigation of therapists’ behavior by addressing the following three research questions:

(1) What strategies did therapists adopt and how did they apply these strategies in expressing agreement in the therapeutic conversation with the Chinese autistic children?
(2) What were the sequential positions of agreement expressions in the therapeutic conversations?
(3) What intervention functions did agreement expressions perform in the conversations?

## The Present Study

### Collection of Conversation Data

The therapeutic conversations in this study were collected in the Department of Rehabilitation at the Children’s Hospital of Zhejiang University School of Medicine, from July to August 2021. Located in Hangzhou, this hospital is one of the National Clinical Research Centers for Child Health in China. Five therapists working in the center took part in the data collection. All of them had worked as therapists for children with ASD for a period of between 2 and 7 years.

In the center, the therapists use ABA therapy during interventions. As the widely accepted and evidence-based intervention method, ABA places more emphasis on therapists actively engaging with students as well as directing and prompting student behavior (Casey and Carter, 2016). Based on the rubric of ABA, psychologists and therapists have developed types of intervention techniques, such as Discrete Trial Training (DTT), Pivotal Response Treatment (PRT), Early Intensive Behavioral Interventions (EIBI) and Naturalistic Intervention (NI).

Free talk constituted the conversation data of this study, which were collected when the therapists were practicing NI. A distinctive feature of NI is the use of materials and toys that motivate the children to engage in the target behavior and promote the generalization of skills. Compared with DTT, NI offers low-structured activities which the children with ASD are able to select within a specific environment, thus creating more verbal interaction and a more spontaneous display of skills (Franzone, 2009). These skills include language skills, social skills such as learning to play games and cognitive skills. In this study, the activities adopted by the therapists during the intervention included telling stories, drawing pictures, playing games and free talk.

Ten male children with ASD, receiving therapy in the center, were recruited for the data collection, with a mean chronological age of 4 years and 8 months. The children had previously been diagnosed with ASD by the pediatricians in the center based on DSM-5 and the pediatrician’s clinical expert judgments.

To obtain information regarding each child’s strengths and needs, the children were asked to receive the Psychoeducational Profile Third Edition (PEP-3) assessment. PEP is regarded as an appropriate tool for planning individualized educational programs for children with ASD (Schopler et al., 1989). There are three composites and 10 subtests in PEP-3. The three composites are the Communication Composite, the Motor Composite and the Maladaptive Behavior Composite. The Communication Composite is a composite of the Cognitive Verbal/Preverbal, Expressive and Receptive Language subtests, the score of which indicates the development level of cognition and language (Schopler et al., 2005). In this study, seven of the 10 children were reported as having a moderate level in the Communication Composite, which indicates that their level of cognition and language development is between the severe and the mild level. Two of them were reported as having an adequate level in this Composite. The remaining child (Lele) did not take part in the PEP-3 assessment, as he was transferred to another rehabilitation center by his guardian in Sept. 2021, before the assessment being implemented. Table 1 presents the basic information of the children with ASD.

**TABLE 1:** Basic information relating to 10 children with autism^1^.

No.	Name	Age (month)	PEP-3 (M: Moderate; A: Appropriate)
1	Lele	29	N/A
2	Kuaikuai	36	A
3	Beibei	38	M
4	Pengpeng	58	M
5	Qiangqiang	61	M
6	Mingming	61	M
7	Hanghang	62	A
8	Haohao	68	M
9	Hengheng	74	M
10	Anan	78	M

*^1^To protect the privacy of the children, all of them were given a pseudonymous name in the study. In addition, the age of the children refers to the age when the conversation data were collected. With the exception of Lele, the other eight children received the assessment between August 2020 and October 2021.*

The data collection was facilitated by therapists using audio recordings. Before collection, the therapists obtained informed consent from the guardians of children with ASD. The length of the audio recordings used in this study amounted to 7 h and 17 min and the verbatim transcription of the audio recordings totaled up to 62,467 Chinese characters. Table 2 shows the time length of the audio files and their transcriptions.

**TABLE 2:** Time length of the audio files and their transcriptions.

No.	File name	Time span	Number of transcribed Chinese characters
1	Lele	32 min 26 s	3964
2	Kuaikuai	43 min 04 s	6521
3	Beibei	30 min 05 s	2044
4	Pengpeng	23 min 39 s	3991
5	Qiangqiang	39 min 47 s	3855
6	Mingming	64 min 44 s	9056
7	Hanghang	93 min 29 s	14, 340
8	Haohao	33 min 06 s	5266
9	Hengheng	26 min 44 s	5688
10	Anan	50 min 47 s	7742

### Research Method

In this study, agreement is defined in a broader sense, i.e., as the speech acts by which the therapists demonstrate their acceptance of the linguistic expression, behavior, affect and attitude of children with ASD in relation to their prior conversation turns. Although agreement acts can be realized by verbal or non-verbal means or both simultaneously, this study focused on the therapists’ verbal expression of agreement.

This study adopted conversation analysis (CA) to describe the therapists’ agreement expressions. As a widely used method in the field of human interaction, CA has been extensively applied in research into psychotherapy since 1960, to provide a thorough description of how psychotherapists accomplish their tasks through talk (Pamela, 2013). To implement this research, we firstly recorded and transcribed the conversation data according to Jefferson’s transcription rules (Jefferson, 1984). In the transcribing process, firstly, we did a verbatim transcription^1^ of all the audio files, and then we provided the first transcription line for each Chinese character by using the *Pinyin* phonetic transcription system and marked the suprasegmental features in *Pinyin*. Below the first line of the *Pinyin* transcription, the verbatim translation of each Chinese word was presented and the third line was the literal translation. To ensure the quality of translation, we discussed with two native speakers of American English.

Pitch, intonation, pause, etc. in conversation were marked during the transcribing process, contextual information was also added in the transcription. These suprasegmental features and contextual information were taken into consideration when we judged whether the expression was indicating agreement or not. Based on the transcription, this study analyzed the phenomenon on two levels, that was the level of action in which the forms and the intervention functions of agreement expressions performed during NI, and the level of sequential organization in which the therapists’ agreement expressions occurred.

## Therapists’ Strategies of Expressing Agreement

Based on the observation of the conversation data, this study identified that four strategies were used to express agreement during the therapeutic conversation between the therapists and the children with ASD. These four strategies are acknowledgment, positive evaluation, repetition and blending.

### Acknowledgments

Bercelli et al. (2008) defined acknowledgment as responses displayed by an individual, demonstrating that they have heard and understood the previous conversation without agreeing or disagreeing with it. However, acknowledgment in this study is mainly defined as affirmation of the previous utterance.

In the therapeutic conversation of this study, the therapists often expressed their acknowledgment in relation to the children’s utterances. The acknowledgment markers used by the therapists included “*dui, duile, hao, haode, en, shide*,” etc.

**Extract 1**

**Table:** 

1	Child:	↑qizhong	yi	ge(.)	↓shi	bu
		LOC	one	CL	is	not
		yong	tu:(.)	disi	ceng
		need	paint	fourth	layer
		‘There is one layer that does not need to be painted, the fourth layer.’				
2	shi	YINWei(.)	naiyou	benshen:	jiu
	is	because	cream	itself	ADV
	shi	bai:se:	de
	is	white	NOM
		‘Because the ice cream is white.’				
3→Therapist:		hao	de
		ok	NOM
		‘Ok.’				

In Extract 1, the therapist and the child were drawing a picture of a drink together. When drawing the fourth layer of the drink, the child proposed to put an ice cream popsicle in it and suggested that this layer did not need to be painted, because both the ice cream popsicle and the paper were white. The therapist gave affirmation of the child’s answer. In this example, “*haode*” (ok) reflects the therapist’s positive feedback in relation to the child’s answer. In addition, this indicates that the child can either continue the conversation or start a new sequence (Wang et al., 2010).

### Positive Evaluation

A positive evaluation expression is similar to the upgraded agreement (Pomerantz, 1984). The difference between these two terms is that Pomerantz’s “upgraded agreement” is relative to the assessment in the prior turn, while the therapist’s positive evaluation functions as a strong agreement with the child’s actions, as well as his/her verbal expressions.

In spoken Chinese, positive evaluation terms include “*henhao*” (good), “*feichangbang*” (very good), “*henbang*” (great), etc.

**Extract 2**

**Table:** 

1	Therapist:	ni	ba	shengxia	de
		2SG	BA-construction	rest	NOM
		bu	yong de(.)	shou
		not use	NOM	put away
		qilai	le
		DV	PRT
		‘You should put away the other toys that you are not using.’			
2	Child:	*^o^*shou	qilai*^o^*
		put away	DV
		‘Put them away.’			
3 →Therapist:		Mingming	zuo	de (.)
		Mingming	do	NOM
		↑feichang	hao
		very	good
		‘Mingming, you did a very good job.’			

In Extract 2, after the therapist and the child finished the game, the therapist instructed the child to tidy up the toys. The child repeated the instructions given by the therapist and carried out the actions at the same time. Subsequently, the therapist praised the child’s behavior in an obvious sharper pitch. In this example, the evaluation conveys not only the therapist’s agreement, but also her positive assessment of the child’s behavior.

### Repetition

Repetition is widely used in daily conversation. As Schegloff (1987, p. 70) stated, “not infrequently in naturally occurring, spontaneous conversation, speakers will repeat, re-say, recycle some part of their utterances.” Regarding function, Kim (2002) revealed that the next speaker’s repetition with downward intonation contour in American English performed interactive functions, such as providing confirmation or showing that the current speaker shared the opinion or agreed with the preceding speaker. In Chinese conversation, the speaker’s repetition indicated similar functions like listenership, alignment, agreement/confirmation, etc. (Huang, 2010).

In this study, we found that the therapists frequently repeated the child’s Second-Pair Part (SPP) to show their listenership, as well as their agreement with the child’s utterance. Following Kim (2002) description of next-turn repetition in American English conversation and Huang’s classification of other repetition in Mandarin child language (Huang, 2010), this study classified the therapists’ repetition into four sub-types: exact repetition, partial repetition, modified repetition and expanded repetition.

Exact repetition refers to “the reproduction of all the words of the model utterance in the same order, without any changes or additions” (Huang, 2010, p. 828), as shown in Extract 3.

**Extract 3**

**Table:** 

1	Therapist:	zhe	liang	ge	yanse
		this	two	CL	color
		yiyang	ma?
		same	Q
		‘Do these two building blocks look the same?’			
2	Child:	bu(.)	yi:yang:
		not	same
		‘They are different.’			
3→Therapist:		bu:	yi:yang: (.)	suoyi(.)	bu
		not	same	so	not
		neng	fang	zai	yiqi
		can	put	PREP	together
		‘They are different, so you cannot put them together.’			

In this example, the therapist asked the child to build a series of blocks. After the child answered her question regarding the color of the block, the therapist indicated her agreement with the child by repeating the child’s answer exactly in a prolonged tone.

Partial repetition, also termed as reduced repetition, refers to an omission of morphemes or content words from the model utterance, as shown in Extract 4. In this example, the therapist and the child were talking about the scorching summer in Hangzhou city. The therapist partially repeated what the child said in the previous turn in an emphatic and prolonged tone.

**Extract 4**

**Table:** 

1	Child:	jianzhi	tai:	re	le
		ADV	so	hot	PRT
		‘It’s too hot.’			
2→Therapist:		TAI:	re:	le(.)	ni
		so	hot	PRT	2SG
		kan(.)	hao	re:	ya
		look	very	hot	PRT
		‘It’s too hot, look, so hot.’			

Partial repetition was used to perform not only an agreement function but also a repairing function. In CA, the term “repair” refers to “practices for dealing with problems or troubles in speaking, hearing, and understanding the talk in conversation” (Schegloff, 2000, p. 207). Addressing the problems produced by the children with ASD, the therapist would initiate and repair the problems by partially repeating the child’s utterance and correcting the mistakes in it.

**Extract 5**

**Table:** 

1	Child:	hua	yi	duo(.)	zui:ba:	
		draw	one	CL	mouth	
		‘Draw a mouth.’				
2→Therapist:		hai	yao	hua	ge	zuiba:(1.5)
		still	need	draw	CL	mouth
		hua	↑yi	↑ge	zuiba:
		draw	one	CL	mouth
		‘We also need to draw a mouth. Let’s draw a mouth.’				

In Extract 5, the therapist suggested to the child that they drew a picture of a fish together. After drawing the tail, body and eyes of the fish, the child found that the picture of the fish still lacked a mouth, and he proposed to draw a mouth. The therapist agreed with the child’s plan and began to draw the mouth. In this extract, the child used the word “*yiduo*” for mouth, instead of “*yige.*” “*Yiduo*” is incorrect because in Chinese, “*duo*” is a classifier used to modify the word flower, while “*ge*” describes items such as person, clock, egg, etc. Addressing the problem, the therapist agreed with the child’s proposal by partially repeating what the child had said and simultaneously correcting the mistake by replacing “*duo*” with “*ge*” in a sharper pitch to attract the child’s attention.

Modified repetition refers to using part or all of an utterance as a model, while changing the pronoun, the order of the elements or the complement, etc. (Huang, 2010), as shown in Extract 6.

**Extract 6**

**Table:** 

1	Therapist:	lele	hai	yao	shenmo?	
		lele	still	want	what	
		‘Lele, what else do you want?’				
2	Child:	wo	hai	yao	↑feiji	
		1SG	still	want	plane	
		‘I also want a plane.’				
3 →Therapist:		ni	hai	yao	feiji	a(.)
		2SG	still	want	plane	PRT
		hao	de
		ok	PRT
		‘You also want a plane, ok.’				

In Extract 6, the therapist and the child were playing with vehicle toys. After asking the child to choose a toy, the therapist expressed her agreement with the child’s immediate choice by repeating what the child said in a modified way, i.e., the therapist replaced “*wo*” (I) with “*ni*” (you) in the repetition expression.

In accordance with Huang (2010), expanded repetition in this study is defined as repetition including one element of the model utterance, and the other element created by the therapist without a preceding model. Due to the poor linguistic ability of children with ASD, they were sometimes unable to express themselves clearly and completely, therefore, when addressing this problem, the therapist added detailed information to the child’s utterance.

**Extract 7**

**Table:** 

1	Child:	↑SHui			
		water			
		‘Water.’			
2 →Therapist:		tamen	zai (.)	shui	limian]
		3PL	PREP	water	inside
		‘They are in water.’			

In Extract 7, the therapist and the child were drawing a picture of fish swimming. The therapist indicated that the fish had been drawn, then the child said “*shui*” (water), suggesting that the therapist should draw water because fish swim in the water. The therapist agreed and gave a complete statement by expanding upon the child’s utterance.

### Blending

Blending refers to the strategy of combing the response turn in the SPP of the adjacency pair, produced by the child, along with part or the whole of the First-Pair Part (FPP) of the pair, produced by the therapist who initiated the request. By blending expressions, the therapist expressed her agreement with the child’s response and provided a more detailed and syntactically complete answer to the initiated question.

**Extract 8**

**Table:** 

1	Therapist:	zenmo	ban? (8)zenmo		ban	
		how	do	how	do	
		ne?(9.6)	ni		kan(.)	
		PRT	2SG		look	
		‘What to do? What to do? Look.’				
2		zheli	you	yi	ge?
		here	have	one	CL
		‘Here is a?’			
3	Child:	wan:
		bowl
		‘Bowl.’			
4→Therapist:		you	yi	ge	wan(0.5)	na
		have	one	CL	bowl	DM
		yao	bu? (.)	ni	ba yinliao?	
		want not	2 SG	BA-construction drink		
		‘Here is a bowl. How about pouring the drink into?’				

In this example, the therapist and the child were drawing the picture of a drink. After drawing the picture, the therapist and the child pretended to taste the drink. Since there was only one straw in the picture, the therapist guided the child to observe the bowl next to the drink in the picture. Under the therapist’s guidance, the child gave the correct answer, then the therapist gave a positive response to the child’s answer by blending part of his question with the child’s response.

## Therapists’ Application of Strategies in Expressing Agreement

In accordance with the aforementioned four strategies, this study found that the therapists applied the strategies in two ways: expressing agreement using a single strategy and expressing agreement using multiple strategies.

### Expressing Agreement Using a Single Strategy

In addition to Extracts 1, 2, 3, 4, 5, 7, and 8, the following extract is another example in which the therapist expressed agreement using a single strategy.

**Extract 9**

**Table:** 

1	Therapist:	[tiane	de	liwu
		swan	ASSOC	present
		shi	shenmo?
		is	what
		‘Which gift did the swan have?’			
2	Child:	shenmo?
		what
		‘What?’			
3	Therapist:	wei
		scar-
		‘Sca-’			
4	Child:	weijin
		scarf
		‘Scarf.’			
5 → Therapist:		↑duiy	la]
		right	PRT
		‘Right.’			

In this extract, the therapist and the child were reading a book about Christmas together. The therapist guided the child to observe which gift Santa Grandpa gave to the swan. Under the therapist’s guidance, the child gave the correct answer. The therapist thereupon affirmed the child’s answer using the strategy of acknowledgment in a sharper pitch.

### Expressing Agreement by Multiple Strategies

In the conversation data of this study, there were two means of expressing agreement using multiple strategies: expressing by two strategies and expressing by three strategies.

Extract 10 is an example of expressing agreement by two strategies. In this extract, the therapist suggested to the child that they drew a picture of a drink together. After the therapist asked the child to tell the color of orange juice, the child answered uncertainly. In this situation, the therapist agreed with the child’s answer in a prolonged tone by using the strategies of partial repetition and acknowledgment simultaneously.

**Extract 10**

**Table:** 

1	Therapist:	na	ni	gaosu	wo(.)	cheng:zhi:
		DM	2SG	tell	1SG	orange juice
		shi	shenmo(.)	yanse	de?	
		is	what	color	NOM	
		‘Then can you tell me the color of orange juice?’				
2	Child:	haoxiang	shi(.)	chengse	de	
		maybe	is	orange	NOM	
		‘It seems to be orange.’				
3→Therapist:		cheng:se:	de(.)	dui:	le:	
		orange	NOM	right	PRT	
		‘Orange, right.’				

Extract 11 is another example of expressing agreement by two strategies. In this extract, the therapist and the child were playing a puzzle game that is, comprising a birthday cake with four layers of colors. After the child finished the first layer, the therapist asked the child to observe the color of the next layer. The therapist confirmed the child’s answer by stressing acknowledgment marker “*dui le*” (right) and a positive evaluation “*hen bang*” (very good).

**Extract 11**

**Table:** 

1	Therapist:	zhe	shi	shenmo	yanse?	
		this	is	what	color	
		‘What color is this?’				
2	Child:	fen:	se:		
		pink	color		
		‘Pink.’				
3 →Therapist:		dui	le	Pengpeng	hen	bang
		right	PRT	Pengpeng	very	good
		‘Right, Pengpeng did a very good job.’				

In addition to expressing agreement by two strategies, expressing by three strategies was also used frequently in the therapeutic conversation, as shown in Extract 12:

**Extract 12**

**Table:** 

1	Therapist:	women	xianzai	yiyang	ma?	
		1PL	now	same	Q	
		‘Do our building blocks look the same now?’				
2	Child:	↑yi:yang:
		same
		‘Same.’				
3→Therapist:		yi:yang:	le(.)	hen	bang (.)	hao
		same	PRT	very	good	ok
		‘Same, very good, ok.’				

In this example, the therapist was playing the block-building game with the child. After the therapist finished the block-building, the child was asked to build the block in the same way. When the therapist asked the child whether their blocks looked the same, the child responded correctly. The therapist affirmed the child’s answer by using the strategies of exact repetition in a prolonged tone, positive evaluation and acknowledgment.

## Sequential Positions of Agreement Expressions

Sequence organization in CA indicates how behaviors or discourse are continued or connected in a coherent, orderly and meaningful way (Schegloff, 2007). As the basic sequence in sequence organization, an adjacency pair refers to two or several turns that are related to one another in conversation. Generally, an adjacency pair consists of the FPP and the SPP; these two parts are produced by different speakers. However, such basic sequences are frequently expanded following the SPP. Two main sorts of post-expansions are identified: minimal and non-minimal. Minimal forms are produced by the speaker of the initiating action, which offer an adequate reaction to the SPP, but this reaction does not itself initiate a new sequence, while the non-minimal forms lead to new post-expansion sequences (Stivers, 2013).

In the conversation data of this study, a therapist’s agreement expressions occurred in either the basic two-part sequence or the post-expansions of the sequence. The majority of sequences with post-expansion were either question-answer sequences or request-response sequences. In the case of a two-part sequence, it was the child who initiated the topic by asking for certain items, then the therapist agreed with his/her initiated action. The child’s initiation of the conversation consisted of putting forward a proposal, asking a question, voicing their opinion or making a request, etc.

**Extract 13**

**Table:** 

1	Child:	[wo	yao	(.) hai:xing:
		1SG	want	starfish
		‘I want a starfish.’			
2 →Therapist:		↓oh (.)	haixing (.)
		DM	starfish
		‘Ok, starfish.’			

In this extract, the therapist and the child were playing with animal toys and fruit toys. When the therapist guided the child to distinguish between different kinds of fruit, the child’s attention shifted and he asked for a starfish in the toy box. The therapist did not refuse the child’s request. Instead, she made a positive response.

When the therapist initiated a sequence, the sequence always involved post-expansions in which the therapist’s agreement expressions occurred as a minimal form.

**Extract 14**

**Table:** 

1	Therapist:	ta	yao	gan	shenmo	ya?
		3SG	want	do	what	PRT
		‘What will he do?’				
2		ta	yao	gei	xiaopengyou	
		3SG	want	give	child	
		men	song ?,			
		PS	send			
		‘What will he give the children?’				
3	Child:	↓song	liwu			
		send	present			
		‘Give presents to the children.’				
4→Therapist:		↑dui le(.)	gei	xiaopengyou		
		right PRT	give	children		
		men	song	liwu		
		PS	send	present		
		‘Yes, he will give presents to the children.’				

In this extract, the therapist asked the child to think about what Santa would give to the children. After the child responded to the question, the therapist firstly agreed with the child’s response in a sharper pitch and then repeated the response. In this case, the structure of the adjacency pair is as follows: the therapist’s request for information (FPP)→the child’s answer to the question as part of a sequence (SPP)→the therapist’s agreement with the child’s answer (F_*post*_). In this extract, the agreement expression is a minimal form of post-expansion produced by the therapist who initiated the act of requesting information. With this agreement expression, the therapist indicated that the child’s response to the requesting action was correct.

## Distribution of Strategies in the Conversation Data

Based on the strategies and their sequential positions discussed in the previous sections, this study annotated the agreement expressions and explored the distribution of both single strategies and multiple strategies in the conversation.

Table 3 shows the distribution of single strategies in the conversation data. Among the four types of single strategies, the strategy of acknowledgment occurred with the highest frequency, while that of blending occurred with the lowest frequency. The further calculation, based on Table 3, showed that 75.65% of single strategies occurred in the post-expansion positions and only 24.35% of single strategies occurred in SPP positions.

**TABLE 3:** Distribution of single strategies in the conversation data.

Single strategies	SPP	Post-expansion	Total	Percentage (%)
Acknowledgment	36	109	145	53.51
Repetition	20	56	76	28.04
Positive evaluation	6	25	31	11.44
Blending	4	15	19	7.01
Total	66	205	271	100.00

Table 4 shows the distribution of multiple strategies in the conversation data. Among the seven types of multiple strategies in the conversation data, the multiple strategies of acknowledgment + repetition occurred with the highest frequency, and that of acknowledgment + positive evaluation ranked the second highest. What should be noted is that the three single strategies within these two multiple strategies appeared in the top 3 of Table 3. Moreover, Table 4 demonstrates that the dual strategies accounted for 91.24%, while the triple strategies accounted for only 8.76% in the data. Overall, an average of 80.37% of agreement expressions occurred in the post-expansion positions, while only 19.63% occurred in the SPP positions.

**TABLE 4:** Distribution of multiple strategies in the conversation data.

Multiple strategies	SPP	Post-expansion	Total	Percentage (%)
Acknowledgment + repetition	30	74	104	37.96
Acknowledgment + positive evaluation	9	69	78	28.47
Acknowledgment + blending	2	56	58	21.17
Acknowledgment + repetition + positive evaluation	/	18	18	6.57
Positive evaluation +repetition	/	9	9	3.28
Acknowledgment + blending + positive evaluation	/	6	6	2.19
Positive evaluation +blending	/	1	1	0.36
Total	41	233	274	100.00

Tables 3, 4 show that: (1) the strategies of acknowledgment, repetition and positive evaluation were favored by the therapists in terms of expressing agreement. (2) Compared with triple strategies, dual strategies were apparently favored by the therapists when expressing agreement and (3) most of the agreement expressions occurred in the post-expansion positions rather than the SPP positions.

## Functions of the Therapists’ Agreement Expressions

The fact that a high percentage of the agreement expressions occurred in the post-expansion position clearly indicated that the therapists had a preference for agreement during the therapeutic conversations. A careful observation of the conversation data showed that in the context of NI, the agreement expressions would help create a supportive therapeutic relationship, serve as a positive reinforcer and implement interventions pertinent to communication skills.

### Creating a Supportive Therapeutic Relationship

The therapeutic relationship is the foundation upon which all therapeutic activities are constructed (Pamela, 2013). By expressing agreement with a child’s response, the therapists in this study displayed their roles as active listeners, encouragers and followers, thus creating a supportive therapeutic relationship with the children.

Acknowledgment markers such as “*hao*” (good) and “*dui*” (right), and positive evaluation expressions like “*taibangle*” (great) express not only the therapists’ agreement but also their active listenership and understanding, as well as their warmth and encouragement. This supportive relationship can be best demonstrated in cases when the children’s utterances contained incorrect expressions. In this situation, the therapists firstly showed their agreement with the expression and then corrected the mistake, instead of correcting the mistake directly, as shown in Extract 15:

**Extract 15**

**Table:** 

1	Child:	jie:jie:(.)	ni	neng	yiqi
		sister	you	can	together
		wan	ma?
		play	Q
		‘Sister, can you play together?’			
2	Therapist 1:	wo	bu	wan:
		1SG	not	play
		‘I do not want to play.’			
3	Therapist 2:	na(.)	↑suan	le
		DM	mind	PRT
		‘Forget it.’			
4	Child:	suan	le(.)	xiamian	yi:qi:
		mind	PRT	next	together
		wan	ba?
		play	PRT
		‘Forget it, let us play together next.’			
5→Therapist2:		en(.)	↑xiaci	yiqi
		uh	next time	together
		wan	ba
		play	PRT
		‘Uh, let’ s play together next time.’			

In this extract, the child wanted to play games with a pre-service therapist (T1) who was undergoing training in the center. This pre-service therapist refused the child’s invitation. The therapist (T2) seized this opportunity to help the child learn to accept the refusal. When the child made a slip of the tongue in expressing “Let’s play together next time.” by mistaking “*xiaci*” (next time) for “*xiamian*” (under), the therapist did not directly correct the mistake. Instead, she agreed with the child’s ideas at first and then corrected the mistake with a modified repetition.

As a follower, the therapist would deviate from the planned intervention schedules to accommodate the child’s choices. In this way, the therapist created a shared interaction, instead of the interaction being guided by the therapist alone, as shown in Extract 16:

**Extract 16**

**Table:** 

1	Therapist:	dou	wan	hao	le	ma?
		all	play	finish	PRT	Q
		‘Did you finish playing with all the toys?’				
2	Child:	da	laohu
		big	tiger
		‘Big tiger.’				
3	Therapist:	da	laohu	a,	wo	zhao
		big	tiger	PRT	1SG	find
		zhao	kan(.)	da	laohu	huilai
		find	look	big	tiger	come back
		‘Big tiger, let me look for it.’				
4	Child:	da	laohu
		big	tiger
		‘Big tiger.’				
5→Therapist:		oh(.)	laohu	↑huilai	le(.)	dui(.)
		DM	tiger	come back	PRT	right
		‘Wow, the tiger is coming back. Right.’				
6		laohu	huilai	le(.)	kuai	kan
		tiger	come back	PRT	quick	look
		‘The tiger is coming back, look at it.’				
((The therapist found the toy tiger in the box and took it out.))						
7	Child:	laohu				
		tiger				
		‘Tiger.’			
8→Therapist:		en				
		uh				
		‘Uh.’			
9	Child:	shi	de			
		right	PRT			
		‘Right.’			
10→Therapist:		en				
		uh				
		‘Uh.’			
11	Child:	laohu(1.2) wo		yao	laohu.	
		tiger	1SG	want	tiger	
		‘Tiger, I want a tiger.’				

In this extract, the therapist and the child were playing with animal toys. The therapist intended to finish this game by asking, “*Dou wan hao le ma?*” (Have you finished your game?) in line 1, however, the child asked to continue the game by playing with a toy tiger. The therapist chose to show agreement with the child’s choice to continue the game by searching for the toy tiger in the toy box in line 6 and 7 and giving it to the child upon his request. By expressing her agreement, the therapist’s role as a follower provided the child with more opportunities to interact actively. For instance, she provided an opportunity for the child to perform the request act by saying “*wo yao laohu*” (I want a tiger.) in line 11.

### Serving as Positive Reinforcers

As the most important and widely applied principle of behavior analysis, reinforcement or positive reinforcement occurs when a response is followed immediately by the presentation of a stimulus change, which increases the future occurrence of similar responses. In terms of formal properties, the stimuli of positive reinforcement include the edible reinforcer, the sensory reinforcer, the tangible reinforcer, the activity reinforcer and the social reinforcer. Among these reinforcers, tangible reinforcers refer to items such as stickers, trinkets, school materials, slips of paper, etc., while social reinforcers advocate physical contact, proximity, positive attention and praise, etc. (Cooper et al., 2019). The therapists’ agreement expressions functioned as social reinforcers in the intervention, as shown in the following extract:

**Extract 17**

**Table:** 

1	Therapist:	↓kan	yi	xia(.)	↑huluobo	
		look	one	CL	carrot	
		‘Have a look, a carrot.’				
2		↓deng	yi	xia
		wait	one	CL
		‘Wait a moment.’				
3		↑huluobo	shi	shucai	haishi	shui:guo?
		carrot	is	vegetable	or	fruit?
		‘Is a carrot a kind of vegetable or a kind of fruit?’				
4	Child:	shi	*^o^*shuiguo*^o^*
		Is	fruit
		‘It’s a kind of fruit.’				
5	Therapist:	hao(.)	wo	zhidao	le(0.5)	ni
		ok	1SG	know	PRT	2SG
		zhe(h)	ge(h)(.)	kan(h)	yi	xia(.)
		this	CL	look	one	CL
		‘Ok, I got it. You have a look at this.’				
6		zhonglei	bu	shi(.)	hen
		type	not	is	very
		wending	a
		stable	PRT
		‘Your classification of categories is not very stable.’				
7		zuo	zuo	hao(0.5)	dui (.)
		sit	sit	well	right				
		zuo	zuo	hao
		sit	sit	well
		‘Sit nicely, right, sit nicely.’				
8	Child:	changjinglu.
		giraffe
		‘Giraffe.’				
((After that, the therapist guided the child to classify fruits and animals that were irrelevant to the topic of the carrot.))						
....						
9	Therapist:	ayi	yao	shuiguo.
		aunt	want	fruit
		‘Auntie wants fruit.’				
10		dui(.)	wo	yao	shuiguo (3)
		right	1SG	want	fruit
		↑Huluobo	shi?
		carrot	is
		‘Yes, I want fruit. A carrot is?’				
11	Child:	shucai	lei
		vegetable	category
		‘Vegetable.’				
12→Therapist:		dui
		right
		‘Right.’				
((After that, the therapist used the watermelon to assist the child in recognizing the category of fruit.))						
....						
13	Therapist:	women	zai	lai	zhao:	a(.)
		1PL	again	come	find	PRT
		ayi	yao	↑shucai
		aunt	want	vegetable
		‘Let’s look for it again. Auntie wants some vegetables.’				
14 Child:		shucai
		vegetable
		‘Vegetables.’				
15→Therapist:		↑dui	de(.)	huluobo	shi?
		right	PRT	carrot	is
		‘Right. A carrot is?’				
16 Child:		shucai	lei
		vegetable	category
		‘Vegetable.’				
17→Therapist:		en:				
		uh				
		‘Uh.’				

In this extract, the therapist was guiding the child to learn vegetable classification. During the first attempt, the child failed to classify a carrot into the vegetable category. By saying “*hao, wo zhidao le*” (Ok, I got it.) in line 5, the therapist did not point out directly the mistake. Instead, she continued the intervention by asking the child to classify fruits and animals. After a while, the therapist asked the child to classify the carrot again; the child gave the correct response in this trial and the therapist confirmed the child’s response by saying “*dui*” (right) in line 12. After this correct trial, the therapist continued to consolidate the child’s knowledge of carrot classification by asking questions such as “*huluobo shi*?” (A carrot is?) five times, and the child answered correctly each time^2^. The successful trials in this example indicate the repeated intervention, as well as the function of agreement expressions as social reinforcers in NI.

### Implementing Interventions Pertinent to Communication Skills

The strategies of expressing agreement, adopted by the therapist, not only conveyed their recognition of the children’s utterances but also implemented interventions to improve the children’s communication competence, including their language-production skills and social skills, etc.

Regarding language-production skills, the therapists in this study adopted the strategy of partial repetition and modified repetition, as well as blending, to help the children communicate in a correct, complete and detailed way, as shown in the following extract:

**Extract 18**

**Table:** 

1	Therapist:	zhe	shi	yu	de:	shen:ti(.)zai
		this	is	fish	ASSOC	body
		hua	yi	ge	yu	de?
		again draw one		CL	fish	ASSOC
		‘This is a fish’s body. Let’s continue to draw a fish.’				
2	Child:	san:jiao:xing:
		triangle
		‘Triangle.’				
3	→Therapist:	san:jiaoxing:	↑sanjiaoxing	shi
		triangle	triangle	is
		yu	de?	wei:↑ba:
		fish	ASSOC	tail
		‘Triangle, the triangle is the fish’s tail.’				
4	Child:	wei:↑ba:
		tail
		‘Tail.’				

In Extract 18, the therapist and the child were drawing a fish together. After the child drew the body of the fish, the therapist asked the child what to draw next and the child answered “*sanjiaoxing*” (triangle). As a response to the child’s answer, the therapist began to draw the fish’s triangle tail. In this extract, the therapist conveyed her understanding and agreement with the child’s answer through exact repetition and expanded repetition in line 3. During the expanded repetition, the therapist added the information that the triangle he was drawing was the fish’s tail, thus making the expression “*sanjiaoxing*” (triangle) more understandable. In the following turn, the child understood the therapist’s intervention effort and he repeated the word “*weiba*” (tail) given by the therapist in the previous turn.

Apart from the agreement expressions in Extracts 18 and 19, the expression in Extract 5 expressed the therapist’s agreement while correcting language errors in the children’s utterances. In Extract 6, the therapist’s modified repetition provided a model of person shift in the conversation. In Extract 7, the therapist adopted the strategy of expanded repetition to produce complete and understandable expressions. On the whole, when using these agreement expressions, the therapists not only expressed their understanding of the children’s utterance but also provided a model utterance for the children to imitate.

In the conversation of this study, the therapist’s agreement expressions also played a positive role in improving the child’s social skills, as shown in the following extract:

**Extract 19**

**Table:** 

1	Therapist:	ni	you	namo	duo(.)	
		2SG	have	so	much	
		yi	da	kuai	dangao:	
		one	big	CL	cake	
		‘You have such a big cake.’				
2		women	zong	bu	neng ((Yum, Yum))	
		1PL	always not		can
		jiu	zhijie	chi	le	ba?
		ADV	direct	eat	PRT	PRT
		‘We can’t eat it directly.’				
3	Child:	↑wo	qie
		I	cut
		‘I cut it.’				
4→Therapist:		dui(.)	women	yao	xian	qie:kai:
		right	1PL	need	first	cut
		‘Right, we should cut it first.’				

In this example, the therapist and the child were having a free talk. The topic of their conversation was a birthday party. In their talk, the therapist suggested that the birthday cake was a big piece and should not be enjoyed by the child alone. The child responded that he had to cut it into pieces, which was affirmed by the therapist; then the therapist guided the child to learn the social skill of sharing, using the strategy of modified repetition. By replacing “*wo*” (I) in the child’s turn with the inclusive first-person pronoun “*women*” (we), the therapist not only evoked a sense of commonality and a close tie between her and the child, but also explained to the child that sharing is a social routine.

## Discussion

Based on the conversations between the therapists and the children with ASD in the context of NI, this study found that the therapists commonly used four strategies of expressing agreement in the post-expansion positions. The reasons for this finding, as this study proposed, were related to the intervention method of NI.

This proposal is enlightened by the study on eliding agreement in Ong et al. (2021). Ong et al. researched the eliding agreement (the absence of explicit agreement strategies) used by therapists among themselves when implementing Open Dialogue treatments with adults suffering from mental health problems. The discussion in the study shows that the frequent use of eliding agreements can be explained by institutional influences, including Stocks of Interactional Knowledge (SIK) on the way in which Open Dialogue should be implemented. For example, the Open Dialogue approach suggests therapists to understand clients’ utterances or behaviors from multiple perspectives. If therapists agree with one another’s views in the process, this will reduce the need to voice multiple perspectives. In addition, the strategy of eliding agreement rather than disagreement could avoid risking social solidarity between the therapists, which is in alignment with conceptual ideas, such as collaboration and equality promoted in Open Dialogue.

Based on the principles of Applied Behavior Analysis, NI occurs in the context of naturally occurring activities in order to increase generalization and spontaneity, as well as improve the maintenance of the target behavior. By incorporating child choice, various tasks, attempts at rewarding a child, and direct and natural reinforcers, this method creates a non-aversive environment to improve their acquisition of communication skills (Ashbaugh and Koegel, 2021). These features of NI can explain the therapists’ preference for agreement strategies in the conversation data of this study.

Firstly, as part of their SIK of implementing NI, the therapists often agreed with children’s choices during the intervention process, thus creating a supportive relationship with the children with ASD and providing them with more opportunities to communicate. Secondly, the therapists also used combinations of agreement strategies to implement the intervention. Compared with single strategies, expressing agreement by multiple strategies, as shown in Extracts 12–16, allowed the children with ASD to identify the expressions or behavior with which the therapists agreed, thus having an advantage over those providing general agreement (simple acknowledgment or positive evaluation) in reinforcing verbal skills. This advantage of reinforcing verbal skills was preliminarily measured in the quantitative results of a study, comparing the effects of general and descriptive praise when teaching intraverbal behavior to children with ASD (Polick et al., 2012). The results demonstrated the slight advantage of descriptive praise in terms of teaching efficiency. In addition, the advantage was echoed by the comments of one particular therapist in a discussion with the first author of this study:

*“When we express our praise or agreement, we should make our expression specific. If you just tell the child ‘good’ or ‘OK’, the child may not know what “good” or “OK” means. As a result, it is not very likely that the child will continue to improve on this in the future. So, we must agree or praise in a specific way. If a child tidies up, we will praise the child by saying: ‘that’s so good. You tidy up so well’. Simply telling him, ‘You are great!’ is far from enough”*.

The above discussion demonstrates the institutional influence of the intervention method on therapist talk, as highlighted by Ong et al. (2021). This influence also explains why the agreement expressions in the therapeutic context of this study are different from those in the non-institutional settings. Specifically speaking, previous studies on the agreement strategies in the non-institutional settings mainly investigated how the second speaker conveyed their agreement with the assessment made by the first speaker (Pomerantz, 1984; Heritage and Raymond, 2005). Within this particular context, the various types of agreement expressions denoted the second speaker’s approval and their cognitive stances toward the prior assessment as well as their epistemic rights to make an assessment. However, the agreement strategies used by the therapists in this intervention-oriented context primarily function as intervening facilitators. Moreover, due to different therapeutic contexts and identities of the speaker (client or therapist), the functions of agreement expressions in this study are unlike those discussed in Bercelli et al. (2008).

In general, the current study, together with the existing studies on agreement, demonstrates that context influences the practice of expressing agreement in interaction. For therapists, this kind of influence should constitute part of their SIK when implementing a particular method, in order to achieve better intervention outcomes. However, the existing literature on the therapeutic conversation with individuals with ASD shows that the studies in this field focused on the performance of the group, rather than how the therapists conducted their intervention through the “talk therapy.” Considering this *status quo*, the current study uncovered the agreement practice and its patterns of which therapists may be unaware. This may deepen therapists’ understanding of the agreement expressions in relation to their importance and effectiveness in therapeutic conversations, thus helping to improve the quality of intervention for children with ASD. Moreover, the strategies discussed in this study will provide a reference for future studies relating to expressing agreement during therapeutic interactions with children suffering from other disorders or diseases. Such future studies will illuminate how various therapeutic contexts shape the practice of expressing agreement in conversation.

## Conclusion

This study has revealed how expressing agreement operates and contributes to autism intervention. The analysis found that the therapists employed four agreement strategies, namely, acknowledgment, positive evaluation, repetition and blending. These strategies were used either individually or in combination. The distribution of the strategies showed that most of the agreement expressions appeared in the post-expansion position of the sequence, and these expressions could help create a supportive therapeutic relationship, serve as positive reinforcers and implement interventions pertinent to communication skills. Moreover, this study discovered that the therapists’ preference for agreement expressions in the intervention process could be explained by the features of NI.

As for the limitations of this study, expressing agreement in therapeutic interaction is a multimodal act, which involves not only the verbal mode but also the non-verbal mode, such as nodding, gaze, gestures, smiling, etc. However, this study analyzed solely the verbal mode of expressing agreement. A multimodal study would certainly expand the semiotic landscape of the act of expressing agreement and offer more insights into the intervention practice of therapists. In addition, following the research design of Polick et al. (2012), the effectiveness of the agreement strategies can be compared in intervention sessions within a multiple-baseline design in future studies.

## Data Availability Statement

The raw data supporting the conclusion of this article will be made available by the authors, without undue reservation.

## Ethics Statement

The studies involving human participants were reviewed and approved by The Research Ethic Board of Children’s Hospital of Zhejiang University School of Medicine. Written informed consent to participate in this study was provided by the participants’ legal guardian/next of kin.

## Author Contributions

XZ designed the study, analyzed the data, and wrote the manuscript. The corresponding authors BM and CL conceived this study, collected the data, and revised the manuscript. CL, LZ, and HL transcribed the conversation data, reviewed the literature, wrote sections of the manuscript, and revised the manuscript. All authors contributed to the article and approved the submitted version.

## Conflict of Interest

The authors declare that the research was conducted in the absence of any commercial or financial relationships that could be construed as a potential conflict of interest.

## Publisher’s Note

All claims expressed in this article are solely those of the authors and do not necessarily represent those of their affiliated organizations, or those of the publisher, the editors and the reviewers. Any product that may be evaluated in this article, or claim that may be made by its manufacturer, is not guaranteed or endorsed by the publisher.

## Footnotes

1 The transcription symbols are listed in the Appendix.

2 Due to limited space, this study provided the transcription of the first three occasions.

## Appendix: Transcription symbols

**Table:**

	the end of an overlapping speech
?	the sharper pitch in utterance
↓	the lower pitch in utterance
?	rising intonation, not necessarily a question
?.	a rise stronger than a comma, but weaker than a question mark
word	underline indicates speaker emphasis
(.)	a micropause
?	draw attention to features of talk that are relevant to the current analysis
(())	the transcriber’s descriptions of events
(7.6)	numbers in round brackets measure pause in seconds
CAPITALS	mark speech that is louder than surrounding speech
,	a continuing intonation
.	mark falling, stopping intonation
:	prolongation of prior sound
^°^word^°^	the talk is softer than the talk around the surrounding
(h)	laughing sound in utterance
